# The social and sexual lives of Black sexual minority men 30 years of age and older in South Africa

**DOI:** 10.1186/s12889-022-14303-5

**Published:** 2022-10-15

**Authors:** Amy Crandall, Happy Phaleng, Jagadīśa-devaśrī Dacus, Oshin Bista, Pierre Brouard, Dawie Nel, Vasu Reddy, Theo Sandfort, Justin Knox

**Affiliations:** 1 Mailman School of Public Health, Department of Sociomedical Sciences, Columbia University, New York, NY USA; 2 OUT LGBT Well-Being, Pretoria, South Africa; 3 Institute for Sexual and Gender Minority Health and Wellbeing at Northwestern University, Chicago, IL USA; 4 New York State Psychiatric Institute, HIV Center for Clinical and Behavioral Studies, New York, NY USA; 5 Department of Sociology, Faculty of Humanities, University of Pretoria, Pretoria, South Africa; 6 Department of Psychology, Faculty of Humanities, University of Pretoria, Pretoria, South Africa; 7 Department of Psychiatry, Columbia University Irving Medical Center, New York, NY USA

**Keywords:** Sexual minority men, South Africa, Stigma, Sexual orientation, Aging

## Abstract

**Background:**

Black sexual minority men (SMM) ages 30 and older are under-represented in HIV studies in sub-Saharan Africa, despite being at increased risk of HIV infection and contributing to potential onward HIV transmission. To better understand the social and sexual lives of older Black South African SMM, we conducted in-depth interviews with SMM who were > 30 years old.

**Methods:**

From March–September 2016, we recruited a convenience sample of 37 SMM ages 30 and older by partnering with an LGBTQ+ organization in Tshwane, Pretoria. Men were interviewed about various aspects of their lives, including their sexual orientation, social connectedness, experiences with stigma and perspectives on participating in research.

**Results:**

Participants described their experiences with their sexual identities, cultural and social implications of disclosure, and their perspective on South Africa’s political perspectives on the LGBTQ+ community. Men described how these experiences influence their trust in research and comfort participating in studies.

**Conclusions:**

Inferences drawn from these findings provide direction on how to improve middle-aged SMM’s representation in research, such as recruiting a higher proportion of older and middle-aged SMM to serve as seed participants and building stronger community partnerships to disseminate study findings to settings where data collection is conducted.

**Supplementary Information:**

The online version contains supplementary material available at 10.1186/s12889-022-14303-5.

## Background

In sub-Saharan Africa, gay, bisexual, and other sexual minority men (SMM) are disparately impacted by the Human Immunodeficiency Virus (HIV) [1–9]. A systematic review and meta-analysis estimated the overall HIV prevalence among SMM in sub-Saharan Africa to be 18%, nearly five times higher than men in the general population [10]. Social stigma in sub-Saharan Africa has created a climate where SMM often remain hidden, which contributes to an increased risk of HIV transmission [10], and makes it more challenging to researching these populations.

Despite these obstacles, researchers have reached pockets of SMM in sub-Saharan Africa for study inclusion. Early HIV prevention studies relied on convenience sampling to recruit SMM [11–13]; however, recent efforts have centered on respondent driven sampling (RDS), a chain referral system in which recruitment is systematically tracked [14]. Once statistical weighting is performed [15, 16], RDS samples are theoretically generalizable to an underlying study population [17]. However, a common limitation among studies of SMM in sub-Saharan Africa is the low number of middle-aged and older SMM (SMM age > 30 years) included in their sample populations. For example, the average median age was 23 years across studies among SMM in sub-Saharan Africa that used RDS, and no study reported a median age greater than 30 years [18–33]. In RDS studies conducted among SMM in South Africa, 70% of SMM were younger than 25 years [3, 5, 22, 34, 35], despite men this age and younger comprising 23% of the male population in South Africa [36].

Consequences to the limited representation of middle-aged and older SMM in sub-Saharan African HIV research underestimates the extent of the HIV epidemic within this population. Not only are older SMM at increased risk of HIV infection [3, 22, 34, 37], but also they may perpetuate the cycle of onward transmission because older SMM experience higher sexual vulnerability to HIV [38]. A mathematical modeling study estimated that the median HIV prevalence among SMM in South Africa has been underestimated by 8.5% as a result of the underrepresentation of SMM ages 30 and older in seroprevalence studies [33]. This underestimate prevents necessary resources (e.g., funding, healthcare provision) from being allocated to effectively address older SMM’s unique HIV prevention needs. Provide much needed HIV Furthermore, research among these populations are novel as we were unable to identify any previous studies that focused on older Black African SMM in sub-Saharan Africa, including any qualitative studies. Therefore, the objective of the current study is to investigate why Black South African SMM who are 30 years and older are not included in research studies. Because the median age of participants in all of the previously identified studies was less than 30 years of age [18–33], we focused on SMM who were at least 30 years old.

In order understand why Black South African SMM who are 30 years and older are not included in research studies, we decided to ask them about their social and sexual lives, and thus we explored the following issues in our interviews with them. First, we discussed sexual orientation, including sexual identity, sexual attraction, and sexual behavior. We asked about these various aspects of men’s sexual orientation because we recognize that these domains often do not converge, particularly in settings where sexual minority groups are highly stigmatized [39]. While exploring these topics, we also wanted to account for the fact that sexual identity development is a fluid process for many SMM [40–48], so we asked SMM about their current sexual orientation and about their sexual orientation in the past. Another topic that we explored with SMM was their social connectedness to other SMM, such as how they describe their community or communities, how they relate to these communities, where they find social support, if they are connected to Lesbian, Gay, Bisexual, Transgender, Queer/Questioning Plus (LGBTQ+) organizations, and to other younger SMM. We also asked SMM about the impacts of stigma on their lives. As seminal work on stigma [49], including Sexual Minority Stress Theory, [50, 51], have described, stigma includes both external acts of discrimination enacted upon individuals who engage in same-sex sexuality (i.e. external stigma) and the internalization of one’s stigmatized status (i.e., negative feelings and thoughts about one’s sexual orientation among individuals who experience same-sex sexuality). Therefore, in the interviews, we asked SMM about experiences of external stigma, perceived norms about gender roles, experiences conforming to these norms about gender roles, and about internalized homophobia [49]. Sexual Minority Stress Theory has also shown that stigmatized individuals experience health disparities and limited access to care, so we also asked SMM about experiences with discrimination in healthcare settings [49–51]. Lastly, we asked SMM about their previous participation in research, which we did not expect them to have participated in research before, or to have formulated explicit opinions about reasons for this, or for why older SMM are often under-represented in research. But we at least wanted to explore their awareness of research and ask if they had previous experiences with or attitudes towards research. Collectively, we anticipated that through discussions on these topics with SMM ages 30 years and older that we could learn about their social and sexual lives, providing insight as to why they have been under-represented across research efforts, thus far.

## Methods

### Participants

Recruitment for this study was conducted in collaboration with OUT LGBT Well-Being, an LGBTQ+ organization based in metropolitan Tshwane (Pretoria), South Africa. OUT staff members identified potential candidates at their facilities and in areas where recruitment could be conducted confidentially. Recruitment flyers were also distributed at OUT facilities, gay-friendly commercial and social venues, and at healthcare facilities that provide services to SMM. Additional referrals were made by interview participants. Those who were eligible were [1] at least 30 years old; and [2] reported having ever had oral, anal, or masturbatory sex with at least one man, regardless of their sexual identity. Participant recruitment and data collection were conducted from March to September 2016. A total of 37 men were interviewed for the study. The study protocol was approved by the Institutional Review Boards at the New York State Psychiatric Institute (New York, New York, USA) and the University of Pretoria Faculty of Humanities (Pretoria, South Africa).

### Procedures

Interviews were conducted by a trained Black male interviewer using a semi-structured guide. The interviewer was trained to systematically ask the questions in sequential order for each interview and to probe for more in-depth information. Because probes were not standardized, the interviewer rehearsed asking probing questions. We collected participants’ demographic and background information (i.e., social support, sexual attraction and identity, gender expression, internalized homophobia, disclosure, discrimination, alcohol use, and mental health using a quantitative assessment. These were followed by qualitative items on a variety of topics including sexual identity and attraction, disclosure of orientation, sexual behavior, concerns regarding HIV and general safety, community connectedness, changes over time in acceptance of same-sex sexuality, and healthcare experiences (see Table 1 for the complete qualitative interview guide). Interviews lasted approximately 60 minutes. Interviews were audio-recorded and conducted in private areas at the central Pretoria offices of OUT or in a private and secure space in one of the four townships (e.g., healthcare facilities that treat SMM or participants’ homes), depending on the participants’ preference. All interviews were conducted in English, although participants interspersed other languages (Tswana [Setswana], or Northern Sotho [Sepedi]), as is common in conversations with Black South Africans. All interviews were transcribed verbatim. Quotes presented in the results were either originally in English or translated into English and presented verbatim. In accordance with Guest et al. (2006), who found that after 12 interviews, 92% of codes were identified, we posit data saturation with our 37 interviews [52].

**Table 1:** Interview guide (sections and sample items) for in-depth interviews among *N* = 37 Black sexual minority men in Tshwane (Pretoria), South Africa (March–September 2016)

Background information	
Sociodemographics (e.g., age, living situation, education, employment, income, family)	
Sexual and Gender Identity	
How do you identify yourself in terms of your sexuality, attraction	
Do you feel that perceptions of MSM have changed over time	
Concerns	
What specific concerns do you have in your life as a man who has sex with men (probe for health-related concerns, including perception of risk of HIV infection)?	
What do you do to address these concerns (probe for safer sex practices and HIV testing, if mentioned)?	
Links to Other SMM and the Community	
How are you linked to other gay men?	
Are you linked to MSM/LGBT organizations – in what way?	
Community	
How would you define your community/ies?	
How do you describe your relationship to your community/ies?	
Constitution	
Are you aware of what the South African constitution says about same-sex sexuality?	
Did that change make an impact on your life?	
Healthcare	
What do you see as facilitators/barriers for accessing/utilizing healthcare?	
Discrimination	
Have you had experiences with discrimination/stigma/social pressure?	
Participation in Research	
What is your experience with research studies like these?	
What do you see as facilitators/barriers for participating in research studies like these?	
Conclusion	
Other information that you want to share?	

### Analysis

Coding of the in-depth interview transcripts was completed in multiple stages using rigorous, previously validated methods [53, 54]. First, concept-driven codes were created using the study’s main research questions and the interview guide. This was followed by data-driven coding using a random selection of multiple interview transcripts. The qualitative codebook for interviews was tested and refined by using it to code a random selection of multiple interview transcripts. Initially, the random selection of multiple interview transcripts was coded by two coders independently, who then discussed and agreed upon applications of the codes and produced reconciled, coded transcripts. Once a full consensus was met, the remaining transcripts were coded independently by the two coders. Coders met with the primary investigator intermittently during the coding process to discuss relevant issues and arrive at a consensus regarding the application and interpretations of the codes to reach 100% inter-rater reliability. The codebook contained the following codes: Sexual Identity Development, Sexual Attraction Development, “Born This Way,” Internalized Homophobia, Meeting partners, Alcohol Use, Leisure Activity, HIV Prevention and Concerns, Relationships, Links to other SMM, Social Support, Healthcare Utilization, Family Attitudes, Stigma, Discrimination, Disclosure, Gender Norms, Changes over time in Perception of SMM, and Legal Acceptance. DeDoose Version 8.0.35 was used for all coding and data processing. All participants are referred to by pseudonyms to ensure anonymity.

## Results

### Participant characteristics

From March through September 2016, our study team interviewed 37 Black South African SMM. Sociodemographic and psychosocial characteristics of participants are presented in Table 2. Most men (92%, *n* = 34) were in the 30–39 age range, with one participant in his 40’s, and one in his 50’s. Most men (81%, *n* = 30) identified as gay. The majority of participants (62%) reported a regular source of income (89%) not being married. In addition to demographic information, participants were asked about their femininity, social support, internalized homophobia, openness with identity, and experiences with violence using scales from a previous study among Black sexual minority men in South Africa [5]. Table 2 presents the results of the quantitative questionnaire. All scales used had good to excellent reliability: femininity (α = .809), social support (α = .745), internalized homophobia (α = .754), openness with sexual identity (α = .897), and experiences with violence (α = .931).

**Table 2:** Sociodemographic characteristics and psychosocial factors of *N* = 37 Black sexual minority men in Tshwane (Pretoria), South Africa from March–September 2016

	N	%
Age		
30–39	34	91.9
40	2	5.4
50	1	2.7
Sexual Orientation		
Gay	30	81.1
Bisexual	5	13.5
Straight	1	2.7
Other	1	2.7
Regular Income		
Yes	23	62.2
No	13	35.1
Married		
Yes	2	5.4
No	33	89.2
Other	2	5.4
Education		
Primary	2	5.4
Secondary	14	37.8
Higher	21	56.8
	Mean ^a^	Std. Deviation
Femininity ^b^	2.9	.76
Internalized Homophobia ^c^	1.9	.53
Social Support ^d^	3.7	1.13
Openness of Identity ^e^	3.1	.80
Experiences with Violence ^f^	1.5	.63

^a^All of the scale response options range from low agreement with statement [1] to high agreement with statement [5], thus higher scores represent higher levels of the respective construct

^b^Femininity measures self-reported presentation of femininity in their appearance, interactions, and behavior

^c^Internalized homophobia measures self-reported outlook on their identity and desire to change one’s identity

^d^Social support measured self-reported levels of social, financial, or medical support within one’s community or family

^e^Openness of Identity measured self-reported willingness to disclose one’s identity to community members, coworkers, or family members

^f^Experiences with violence asked about experiences with physical or sexual violence, as well as gender- or sexuality-based discrimination

### Sexual orientation

When asked how they identify in terms of their sexuality, participants used several different terms, including gay, bisexual, bottom, and a man who has sex with men. Other participants did not label their LGBTQ+ identity and instead stated that they are sexually attracted to other men*.* When participants discussed their experiences becoming aware of their same-sex attraction, most men described becoming aware of their same-sex attraction from a young age. One participant shared that his feelings “*started to become strong*” during primary school. Lutendo, a gay identifying man in his 30’s, recalled his concerns at school, and described how he “*didn’t want to share a toilet with boys because I felt somehow [different] and even when they touched me, I still felt different.”* Participants shared some of their thought processes as they began to realize their sexual attraction. For many, socializing with family or community members who were gay, or encountering media depictions of same-sex relationships, helped them to identify what they were experiencing. Moeketsi, a man in his 30’s who identified as gay described how he learned about same-sex relationships:

[The feeling] has always been there as a kid, and I think it differs from person to person. I am a reader, so I have read a lot about this, and I have always known it was there and it’s not something that shocked me. I always had information and I was at a boy’s school, so it was actually a mission to hide it from my friends to avoid bullying.

As Moeketsi indicates, and other participants also expressed, it was difficult to come to terms with their same-sex sexuality while growing up. Some men felt pressure to keep their identity hidden due to the close nature of their families or communities and fears that they would not be accepted. Many participants thought that people in rural areas were less accepting than in urban areas. Danisa, a man who grew up in a rural part of Zimbabwe shared a poignant experience from his childhood:

Growing up in the rural [area] and bathing in the river, everyone would be naked, and you will be looking at everyone like, ‘nice butt, nice dick, etc.,’ and you start imagining things and end up not bathing, and just saying you have forgotten something somewhere else. You are running away because you’re horny and embarrassed in front of everyone.

Some participants discussed femininity and said that they felt like women, often describing their desire to perform femininity through clothing or actions. Several participants referred to themselves as the woman in the context of sexual partnerships, which they equated with being bottoms or the receptive sexual partner. When describing what it was like growing up, Lenka, a man in his 30’s, explained:

Growing up, the family always identified me as a man, but deep down I have always felt like a woman. I have never played with what boys normally play with, cars, bicycles. I loved playing with dolls, loved cooking. Even if we were playing games with other kids, I always picked being the woman or the mother of the house.

The association of certain behaviors with femininity was mentioned by several participants. As several other participants, Lenka did not use the label transgender when describing their feminine sense of self. While some participants described themselves as females, there were no indications in the interviews that this was indicative of gender dysphoria. It is possible that many of our participants grew up with a binary understanding of gender, sex, and sexual behavior, and identifying as a woman might be a way of integrating their desire for men into a heteronormative frame of reference. It is possible that with greater awareness of transgender identities, some participants may have described gender identities other than male, though this topic was not specifically explored during the interviews.

Many men developed a better understanding of their sexual identity through sexual experimentation. For example, Khutala, a participant in his late 30’s who identifies as gay, discussed one of his early sexual experiences, which took place when he was 16:

I was confused, I didn’t know what was going on. I only knew I was gay, but when it comes to sex and kissing, I was still young, so I didn’t know what was happening. We started like we were joking. He said I am his and he is mine, something like that. Some of our friends saw it and they also said they see we mean something, like a relationship, and we ended up together.

Participants shared that they explored their identity through their sexual debut. For several participants, their first sexual experiences involved an older man, often one who had some level of authority over them. For example, Danisa described:

It was my teacher for me. To realize that he is into men … he was just nasty always sending me to his [house] … rural schools teachers have houses there. We both don't know what happened, I just found myself in bed with him.

Danisa’s story highlights an important dynamic of power and coercion. When probed further, Danisa stated explicitly that he considered this experience to be consensual and not sexual coercion or assault. It is important to note, though, that others might consider it to be sexual violence.

Many of the stories shared with us were used to compare how participants engage with sexuality now with when they were younger. Participants often said that they are now interested in relationships, and that they are not just looking for casual sex. Lenka said this succinctly: “*I would say the only change is that I am no longer sleeping around for experience. I now sleep with someone who I am in a relationship with than previously, when I would sleep with whoever I’m attracted to.”*

Several participants were in monogamous relationships described as positive, equal, happy, committed, and fulfilling. Participants expressed enjoyment with doing activities with their partners, like cooking and traveling. When discussing his current relationship, Sanele, a gay identifying man in his 50’s, explained that he is “that type of person who stays in a relationship. Like, I can mention five years, and three years, and another three years as we’re speaking. I’m in a committed relationship that’s been three years running.”

### HIV and sexually-transmitted infections (STIs)

While describing how their sexual experiences have changed over time, some participants discussed being focused more on sexual health and HIV prevention while aging. Rendi, a man in his 30’s who identifies as gay, said he felt more “confident” about using condoms, which he believed led to him having stronger connections in his sexual relationships. Other men discussed feeling more experienced with safer sex and HIV prevention with age. When asked broadly about what concerns they had in their life, many participants answered that HIV was their main concern. Participants were not explicitly asked about their HIV status during the interview, although four participants disclosed living with HIV. Many men also mentioned acquiring STIs as a concern. Khutala elaborated on this, including why he feels it is a challenge to practice safer sex in a relationship:

My concern is about safe sex and all that, because it’s not easy to use a condom. It’s so easy to not use it. Another thing is especially when you have sex with one person over and over again, because you would feel like you know each other better. I have had sex with a couple of people, but I would usually check myself out if I am still HIV free because it’s so easy not to use protection, even with my current partner, now that we’ve been dating for 4-5 months. We have had sex on three occasions without using protection. That is a lot of the concern for me.

### Disclosure of same-sex attraction or sexual orientation

Many participants reported disclosing their same-sex attraction or LGBTQ+ identity to their families. Some families were supportive and accepting. For example, one respondent shared his process of learning what it meant to be gay, and after telling his sister that they felt something was wrong with them, his sister responded “*there is nothing wrong with you, it’s just that you do things differently*.” Other men shared stories of family members that subsequently got involved with the LGBTQ+ community on their behalf. Some shared stories of seeing Pride events and hearing supportive comments from their families. After coming across a parade while out shopping, one participant’s mom suggested they follow along with the parade.

My mom had good time and she was walking with this other tall drag queen. I was not following their conversation but at the end she said, ‘it's tough at the top darling,’ and I don't know what she meant by that and my mom kept on using that always … She said, ‘My child, where is your girlfriend? Or is it tough at the top darling?’

Not all families were accepting and supportive. For example, Masopha, a gay identifying man in his early 30’s, described the following experience with his family:

When the stage came where I was dating other men, I realized that my relationship with my father was damaged for good. Yes, my father told me that he will never do anything for me because in his family we don’t give such things, and I am no longer part of his family. So that was emotional. I was not that comfortable in my home.

A fear of rejection was often cited as a reason why some men had not disclosed their same-sex sexuality to their families. Even for those who had been open about their same-sex attraction, many of them described how difficult it was. An overwhelming majority of the participants felt that their family members’ negative reactions to their sexual identity was influenced by religion. The notion of same-sex attraction being related to demonic behavior or possession was mentioned by several participants. Kabelo discussed his experiences coming from a very religious household:

I have had a very hard experience when it comes to same-sex sexuality … I’m from a very Christian family, and society also doesn’t accept it, which has made my life very difficult … They don’t agree with my lifestyle. I come from a Christian background, and two of my brothers are pastors. With that being said, being gay is a serious problem. We had a clash … to think that my family told me I’m going to burn in hell, I know my God still loves me.

Disclosure was also mentioned as an important part of many men’s friendships. Most men had disclosed their sexual orientation to their friends and emphasized the importance of having friends to trust and be comfortable with. A few men discussed not disclosing their sexual orientation to their friends or having friends who did not disclose their sexual orientation to participants. This was presented as a barrier to friendship and intimacy. Some participants said they exclusively socialize with other gay men, while other participants had more mixed sexual orientation friendships, such as heterosexual friends. Some described losing friends after disclosing their sexual orientation d. A fear of social isolation was prominently expressed during interviews, and this was a factor in participants’ decision-making process for sexual orientation disclosure. Ntsumi, a gay identifying man in his early 30’s, told us about his friends “*[leaving] the friendship circle*” after Ntsumi came out to them: “*Some supported me where others chose to discriminate*.”

### Social connectedness

Participants were asked to define their community or communities. While most men understood “community” as their friendships with other gay men, others talked about being linked to LGBTQ+ organizations. Almost all men said that being social and connecting with others was important to them. Khutala stated that the people he goes out with often are his “*best friends from high school, we’re still together and we see each other every month*.”

For most participants, drinking establishments (e.g., pubs, clubs, taverns) are places where they go to socialize with friends, relax, meet new people, and meet sex partners. A few men discussed preferring to socialize without drinking alcohol. When discussing alcohol in social settings, multiple participants focused on buying drinks for other men or being bought drinks as a way to attract potential partners. Participants explained that this is something that can create complicated dynamics regarding expectations around sexual behavior. Rendi stated: “I do not ask people to buy for me because they always want something in return, so I [bring] my own money so that I can go home in peace and relax.”

Beyond drinking establishments, some men described meeting sex partners online and through social media. One participant mentioned feeling more comfortable meeting people online. He liked it because it was easier to stop communicating with a potential partner if it wasn’t working out. Rendi elaborated on this as well: “*We communicate and set a date and meet, and if you are not my cup of tea, I would tell you that ‘*you are not my cup of tea, sorry.’” Others shared that social media made it easier for them to be more confident, stating: “*I prefer meeting on social media first because in person, I am kind of shy.”*

Social media was also discussed as a way to connect with and make new friends. Mamello (early 30’s, identifies as gay) shared that: “*On social media, I’ve got brothers [friends] for life*.” Several men discussed perceived generation gaps between them and younger SMM, particularly regarding the use of social media. For example, Rendi described: “*Younger gay men are the most dominating on social media … [they are] more informed than us older gay men, and coming out for them is too easy, because they socialize and communicate with one another*.”

Some men discussed connections to LGBTQ+ organizations while other participants described having no connection to any LGBTQ+ organizations. Participants perceived LGBTQ+ organizations as opportunities to receive healthcare and HIV prevention services. Mosegi, a mid-30’s man who identifies as bisexual, shared how he used a LGBTQ+ clinic: “*If you need condoms, they are there. If you’re sick, there is a doctor, so you can get anything that you could at the hospital, everything is there.*” Others, like Qukeza, an early-30’s bisexual-identifying man, found a social home within these organizations: “*I participate in their activities and workshops. I like OUT because it is based in town and with someone like me who is very secretive, it works well.”* Participants discussed liking meeting other gay men through LGBTQ+ workshops and community activities. A few men discussed volunteering or working for community groups and being engaged in activism.

### Community and internalized stigma

When participants were asked to describe specific concerns in their lives, many men responded not only being concerned about HIV and STI’s, but also about violence and discrimination due to multiple overlapping identities. Participants reported stigma in school, in the community, when accessing healthcare, and at work. Men described instances of discrimination that they encountered due to their sexual orientation and/or feminine behavior. Several participants described anecdotes of being teased or bullied at school by other students or teachers because of their nonconformity to masculinity. One man shared that being teased for spending too much time with girls at school, which led him to hang out with only boys. Now as adults, many men describe experiences of discrimination based on their perceived sexuality.

When asked about where they experienced discrimination, many men shared discriminatory experiences in non-LGBTQ+ focused healthcare settings. When asked about utilizing healthcare, many men discussed issues of confidentiality and discrimination. For many men, healthcare clinics were uncomfortable places. Men described negative experiences with healthcare staff making assumptions about them being HIV-positive because of their sexual orientation or making derogatory comments about them because of their sexual orientation. After Rendi disclosed his sexual orientation to his doctor, he described how the doctor responded by saying: “*We need to pray for you, and I will refer you to this other pastor in town. He is good at this, he can pray for you to get rid of this thing, because it is a demon.*” Due to these experiences, many men discussed avoiding healthcare settings whenever possible, or that they knew of other men who avoided healthcare settings. Men also were afraid their sexual orientation may be disclosed without their consent as a result of seeking treatment. For example, as Amose, a man in his 30’s who identifies as gay, states:

As part of the MSM community, I would say we are vulnerable, and it is hard to access services we end up taking care of ourselves because we are worried that if they see something in our anus, what would they think? Because I’m not even sure if I’m gay, and being at the clinic, I don’t want to give any indication that I’m gay.

Another frequent reason that participants gave for not utilizing healthcare, particularly HIV testing, was a fear of testing positive. Mamello described this stigma and how it discourages people from accessing care: “*Most of us gay men [are afraid], we are contracting infections easily, especially if we don’t condomize. So, most of them are losing their life because they are afraid to contact the clinic because they think people will stigmatize them.*”

A more tacit way that stigma against same-sex sexuality impacted men’s lives was through their perceived expectations about gender roles and social norms. Some men described feeling feminine and behaving femininely, while other men felt that there was an expectation that gay men should behave in a feminine way, even though they did not feel feminine themselves. Other men expressed the opposite; Sizwe, a man in his early 30’s who identified as homosexual, explained that “previously, one had to be feminine to be taken seriously as a gay guy … These days, no need for one to dress up in a certain way to prove a point.” In contrast, several men felt pressured to conform to social expectations of masculinity, like playing with cars or bicycles, and dating or having sex with women, particularly once they reached a certain age. For example, Sanele felt pressured by his friends to date girls when he was younger, which led him to have a girlfriend. Some men were previously or currently married to women and some men had children. When discussing their experiences with women, some men explained that they dated women because of a sexual attraction to women, while others said they dated or had sex with women because they felt pressured to do so. Ntsumi described being raped by a female colleague.

Some girls at work would not believe you are gay or that you are into men only. They would come to you saying this and that, and you end up cuddling with a woman and to find you are getting an erection, so you can’t say you are not attracted. I had sex with a girl last year. My work mate came to my place and trapped me, and she wanted to prove me wrong, that I’m not into men.

In addition, participants expressed internalized homonegativity, such as feeling shame because of their sexual orientation. For example, Kabelo said that he resisted being gay and he described how this contributed to him being socially isolated:

When I was 13 or 14, I met gay people and I chose not to interact or mix with them because this was something I was fighting. Me having to mix with them was going to confirm that I am gay, so I didn’t mix with anyone. I waited until I was mature, 20 or 21, and started friendships and I realized it wasn’t me alone.

### Legal environment

Participants were asked about their knowledge of the South African constitution’s perspectives on homosexuality and if they felt legal changes affectively protect same-sex sexuality. Many participants shared that it had made a positive impact in their lives. When asked about the changes he has seen in the way SMM are treated during his lifetime, Kabelo, a gay identifying man (no age provided), shared a poignant experience:

I remember when I was 14 years old, my brother called me gay, and my mother beat him so bad because gay was a bad word that they don’t expect you to know or use as a child. So, we moved from that. Society is mixed up know, and at least everyone has a gay friend and the TV also teaches people about gays and lesbians, it’s quite a trend now.

Several participants felt that the legal changes have contributed to a safer environment with less violence towards sexual minority individuals. A few participants compared the situation in South Africa to the rest of the continent to highlight the significance of these legal changes. Some men thought that the legal changes had contributed to increased comfort levels among younger SMM because they were not alive to experience South Africa when homosexuality was criminalized. Although participants stated that they did not benefit from these protections while they were exploring their own sexual identity, they were pleased about the positive impact of these changes for younger generations of sexual minority men. Many participants also shared that many of these positive changes have been limited to the legal realm, and that discrimination against same-sex sexuality still exists in South Africa. Kefentse, a man in his 30’s who identifies as gay, shared that despite these changes, he still experiences homophobia: “*The treatment we get from the hospital and the clinic is very bad. All government institutions, even police stations and home affairs offices can be a horrible experience when going there*.”

### Research

Participants were asked about their previous experiences participating in research studies. For most, the present study was their first experience as research participants. Among those who had previously participated in research, many expressed concerns about maintaining confidentiality. Another commonly discussed concern was a lack of communication with participants regarding the results of that research. For example, Amose shared: “*I have participated in a study, and what I didn’t like is that we were never given feedback and we were worried the information was going somewhere. I was feeling used.*”

Similarly, when asked how they felt about researchers, Qukeza replied:

Not good. Because of the research, we do not get feedback. We know research is not concluded, it may not work or fail, but at least they need to keep us posted and report back whether it worked or not … I believe the research studies need to be clear and understood. We need to understand the vision and the mission of that research study, and do a community briefing on that research in the community before conducting the research so people know about it.

Participants expressed a desire for more transparency from researchers and communication with them. Many men also shared concerns about breaches of confidentiality. For example, Bandile, shared:

Gay men are fighting for their safety, their life. Most of the people don’t accept gay people. I can say 90% of men who are gay guys are scared to be exposed. People are scared because they have been humiliated and judged and all that, to a point where they want to protect themselves and don’t want to expose themselves.

Bandile added that people like him who are not scared of being “exposed” should encourage others to participate in research. Masopha shared his perspective, and stated: “*[studies] are very important, because it’s where we get more information. Especially for the research to build up for gay people.”*

Before ending the interviews, participants were asked to share additional thoughts or suggestions with the research team. Several participants made suggestions for ways that the research team could assist the community, which included organizing seminars to share the results of research with participants, working in communities to help improve the treatment of SMM, and providing LGBTQ+ resources, especially in townships. Nku, a man in his mid-30’s who identifies as bisexual, explained:

We understand the work that your organization does, but you guys are not visible in townships, especially in Mamelodi, and gays are not really united in Mamelodi. We need to create spaces to allow people to learn and engaged with each other.

## Discussion

This is one of the first qualitative studies to explore the social and sexual lives of a sample of middle-aged South African SMM. Men shared experiences in this process of discovery about their sexual orientation, as well as disclosing their sexual orientation to others. The men expressed varying levels of social connectedness; some participants were highly social with strong connections to friends, family, partners, and community organizations, while others had fewer social connections. Stigma has an impact on their everyday lives; many fear violence against them and experience discrimination, including in healthcare settings. Some men also spoke directly about their experiences with and attitudes towards participating in research. They expressed frustration with findings not being disseminated among them, and a perceived lack of impact that research has on their lives.

Most men identified as gay with a smaller proportion identifying as bisexual, similar to levels observed in other studies conducted among Black South African SMM [5, 11, 12, 38, 55]. Men discussed experiences discovering their sexual orientation which was in concordance with other research on age of sexual debut among SMM in various international contexts [56]. Many men’s early sexual encounters were with older men, which has also been described previously in other studies [57, 58]. Most men shared that their sex lives are different now than when they were younger. Several men proudly shared that they are now monogamous, and that they no longer engage in casual sex, although they acknowledge that they did when they were younger. Many men reported being in fulfilling relationships, and men who were single often expressed desire for a stable relationship. While sexual risk behavior was not an explicit focus of the interviews, many men brought up condom use when talking about their current sexual behavior. The majority of men said that they thought condom use is important, although some acknowledged that they do not always use condoms, especially in relationships. These may reflect established sexual behaviors and preferences; which was reported in a study among older Black SMM in the US [59].

Participants reported varying levels of social connectedness. Based on the quantitative scale that the men completed, which had response options that ranged in value from 1 to 5, the mean score was 3.7, which falls between “sometimes” or “often” for having someone in your life that you can rely on for various aspects of social support (e.g., if you need money, if you need to talk about your problems). The majority of participants indicated that they had discussed their sexual orientation with “most” friends, family, and coworkers on the quantitative survey, which was also illustrated in their discussions in the qualitative interviews. Romantic and sexual partners, friends, and family members were identified as sources of support. Men also described being involved with LGBTQ+ organizations, which have been described as important sources of social support in other settings [60]. Men described going out to bars, clubs, and taverns to socialize and meet new people. This suggests that these venues can be used to recruit seeds for referral-based recruitment methods, such as RDS. For example, these men are connected to other SMM who know about their sexual orientation, so they should be reachable through referral-based recruitment methods. Also, previous research has shown that recruiting participants who socialize at multiple venues within a community may be more important than having a large social network for generating valid RDS samples [61]. Some men described friendships with younger SMM, although most men stated perceived differences between themselves and younger SMM. This may be reflective of a perceived generational gap within SMM communities, or reflective of differences in how men perceived themselves now compared to when they were younger. This issue of aging among SMM and generational gaps within SMM communities has not been well studied in sub-Saharan Africa, thus far, but is a topic that merits further investigation.

Almost all men discussed experiencing discrimination based on their sexual orientation, including in healthcare settings, which has also been described previously [62]. Stigma impacted men in that they felt an obligation to conform to social norms and gender roles [63]. Many men knew instinctively from a young age that their sexual orientation did not fit with local cultural perceptions of masculinity, which is in line with other research [64], particularly among Black communities where there are high levels of stigma towards same-sex sexuality [65–67].

South Africa is exceptional compared to other parts of sub-Saharan Africa in that there are considerable legal protections in place for the rights of LGBTQ+ individuals. South Africa decriminalized male homosexuality in 1994, outlawed discrimination on the basis of sexual orientation in 1996, and legally recognized same-sex marriages in 2006. Many of our participants started to experience their same-sex sexuality while homosexuality was illegal, and many reflected on the impact that these legal changes had in their lives. Despite these legal protections, Black South African SMM still face high levels of stigma and related marginalization [68]. For example, in the South African Social Attitudes Survey, more than 80% agreed that homosexuality was “always wrong”—and young people aged 15–24 were only slightly more tolerant than those over 50 years of age (85%) [69]. However, accepting attitudes towards LGBTQ+ sexuality appear to be increasing in sub-Saharan Africa [70]. For example, in South Africa, two-thirds of the population support keeping the present protections against discrimination on the grounds of sexual orientation in the Constitution. There has also been a ten-fold increase in the proportion of South Africans who strongly agree with continuing to allow same-sex marriage (from 1 to 10%) over the past 10 years [71]. Participants discussed being proud and appreciative of the legal advancements made to protect SMM in South Africa, and reported experiencing low levels of violence on the quantitative survey. Even with these improvements, hate crimes against LGBTQ+ individuals continue to persist in South Africa [72, 73]. Despite experiencing discrimination based on their sexual orientation, participants had relatively low levels of internalized homophobia on the quantitative survey. However, men described attempts to reject their sexual attraction to other men or conceal their sexual orientation from others. This is in accordance with the Minority Stress Theory which posits this as part of the internalization of a stigmatized status [49–51].

Many of our participants had not previously participated in a research study. Among those who had, many shared concerns such the research process being intrusive and expressed a lack of trust in researchers. This has been noted as a concern about research efforts among sexual minority groups in other geographic contexts [74]. Multiple studies have noted the importance of sharing the results of research with the participants [75, 76]. Our findings confirm that this needs to be made a priority area among future research efforts involving middle-aged and older SMM.

RDS and other referral-based sampling techniques are dependent upon a participant’s ability and willingness to refer other eligible participants into research studies. The size (degree), connectedness, diversity, and tie strength within men’s social networks are critical factors impacting recruitment. Many men described having sources of social support in their lives, however, they had not always disclosed their sexual orientation to all members in their social networks. When discussing their relationships with younger SMM, some men described perceived differences, and mostly described meeting them and observing them in bars and clubs, but not in the context of having relationships or friendships. These are likely not the types of relationships that would be conducive to the forms of recruitment that RDS studies rely on. Some men described involvement with LGBTQ+ organizations, while others did not. These observations help to contextualize the referral patterns that have been observed in samples of SMM, which appear to work along age lines, with middle-aged SMM being more likely to refer other middle-aged SMM, and with younger SMM more likely to refer other younger SMM, in accordance with the social network Theory of Homophily [77, 78]. To recruit SMM over age 30 years, researchers can consider including a higher proportion of middle-aged SMM as seed participants, as they will refer other middle-aged and older SMM. As many men expressed involvement with community LGBTQ+ organizations, researchers can consider continuing to partner with them as local collaborators.

Stigma played an active role in participants’ lives. For this, many older SMM may be less likely to participate and refer other participants into RDS studies. Participating in research studies could involve potentially compromising participant privacy and put them at risk of their sexual orientation being disclosed to the individuals that they refer onwards (and possibly further). Future research can explore whether stigma might be experienced differently among middle-aged and older SMM compared to younger SMM. Additionally, the long-term, generational impacts from Apartheid and before South Africa decriminalized homosexuality can be explored. Lastly, SMM with robust social networks may be unwilling to refer other SMM into research studies because of mistrust and dissatisfaction due to previous negative experiences with research. To mitigate this issue, future studies can consider building stronger community partnerships, which include local capacity-building and efforts to disseminate study results in the settings where data is collected. Collectively, the current study has shown that there are multiple barriers to successfully employing RDS and other referral-based sampling techniques to recruit representative numbers of older SMM into research studies.

The current study has several limitations. First, although the study aimed to learn about the lives of older Black SMM, the large majority of participants were in their 30s. We were only able to interview a few men who were 40 years or older. We found difficulty in recruiting Black SMM 40 years and older. Second, while the sample population included participants who identified as transgender or gender nonconforming, we did not include questions surrounding gender identity. Third, our findings are subject to social desirability bias as participants discussed sensitive and personal topics. Lastly, we used a convenience sample for this study, which is not meant to be representative of the entire population of Black SMM in Tshwane, South Africa. However, considerable overlap in responses from the participants indicate that we achieved an acceptable level of saturation with our sample.

## Conclusion

Through this study, we learned about the social and sexual lives of middle-aged (predominantly ages: 30–39 years) Black SMM in South Africa. Although Black SMM are a largely underrepresented population, across studies on HIV among SMM in sub-Saharan Africa [33], they continue to be at increased risk of HIV infection and contribute towards onward HIV transmission [3, 22, 34, 37]. Research focusing on HIV in sub-Saharan Africa should work to ensure the inclusion of older SMM in an effort to make sample populations more representative and more accurately capture the HIV burden [33]. Black SMM discussed their lived experiences as Black SMM, including themes such as sexual orientation, social connectedness, stigma and discrimination, and perspectives on participating in research. Future research can consider recruiting a higher proportion of older SMM to serve as seed participants and building stronger community partnerships to disseminate study findings to settings where data collection is conducted.

## Supplementary Information

**Additional file 1.** Quantitative survey.

## Data Availability

The datasets used and/or analyzed during the current study are de-identified and available from the corresponding author on reasonable request.

## Abbreviations

HIV: Human Immunodeficiency Virus
LGBT: Lesbian, Gay, Bisexual, Transgender
LGBTQ+: Lesbian, Gay, Bisexual, Transgender, Queer/Questioning, plus other sexual and gender minority identities
RDS: Respondent Driven Sampling
SMM: Sexual Minority Men
STI: Sexually Transmitted Infections
