# Supplementary material for: The social and sexual lives of Black sexual minority men 30 years of age and older in South Africa
Source: BMC Public Health. 2022 Oct 15;22:1923. doi: 10.1186/s12889-022-14303-5 (PMC9569126; doi:10.1186/s12889-022-14303-5)
Supplement: Supplementary file 1 — Additional file 1. Quantitative survey. [file 12889_2022_14303_MOESM1_ESM.docx]

**Background**

1. How old were you at your last birthday? ___ ___ years
2. How would you describe yourself in terms of your race?

| *Black/African* | Coloured | White | Indian/Asian |
| --- | --- | --- | --- |

1. What is the main language spoken in your home?

| *English* | Afrikaans | IsiXhosa | IsiZulu |
| --- | --- | --- | --- |
| *Sesotho* | Setswana | Sepedi | SiSwati |
| *Tshivenda* | Xitsonga | IsiNdebele | Other; please specify__________ |

1. What is the highest level of school you attended: primary, secondary, or higher?

| *None* | Primary | Secondary | Higher (university or other post matric institution) |
| --- | --- | --- | --- |

1. How many years of education have you completed? ___________
2. Do you have regular income from work that you are doing (excluding pocket money, grants, etc.)?

| Yes | No |
| --- | --- |

1. Are you legally married?

| Yes | No |
| --- | --- |

1. If yes, is your partner biologically a man or a woman?

| Man | Woman |
| --- | --- |

**Social Support**

Please indicate for the following statements whether for you it is true never; not often; sometimes; usually; or always.

1. There is someone you can rely on in case you need money.

| Never | Rarely | Sometimes | Often | Always |
| --- | --- | --- | --- | --- |

1. There is someone you can rely on to go to the doctor, clinic, or hospital with.

| Never | Rarely | Sometimes | Often | Always |
| --- | --- | --- | --- | --- |

1. There is someone you can rely on to talk to if you have problems.

| Never | Rarely | Sometimes | Often | Always |
| --- | --- | --- | --- | --- |

1. There is someone you can rely on if you need a meal, food, or a place to stay.

| Never | Rarely | Sometimes | Often | Always |
| --- | --- | --- | --- | --- |

1. There is someone you can rely on if you get beaten up, attacked, or hurt.

| Never | Rarely | Sometimes | Often | Always |
| --- | --- | --- | --- | --- |

**Sexual attraction**

The following questions are about how you think about yourself in terms of your sex and your sexuality.

1. Do you currently feel more sexually attracted to men or to women?

| Only to women | More to women than to men | To women and men equally | More to men than to women | Only to men |
| --- | --- | --- | --- | --- |

**Identify as gay**

1. What word would you use to describe your sexuality? Would you call yourself gay, bisexual, or heterosexual, or would you use another word?

| Gay | Bisexual | Heterosexual | Transgender | Other, please specify______ |
| --- | --- | --- | --- | --- |

**Childhood gender non-conformity**

The following questions are about your behaviour as a child, that is, the years “0 to 12.” Please note that there are no “right or wrong” answers.

1. As a child, were your favourite toys and games those that boys or girls played with?

| Only boys played with | Boys played with more often than girls | Both boys and girls played with | Girls played with more often than boys | Only girls played with |
| --- | --- | --- | --- | --- |

1. As a child, did you feel like other boys or more like other girls?

| Exclusively like other boys | Predominantly like other boys | Equally like boys and girls | Predominantly like girls | Exclusively like girls |
| --- | --- | --- | --- | --- |

1. As a child, would you say that you always or never felt good about being a boy?

| Always felt good about being a boy | Usually felt good about being a boy | Sometimes felt good about being a boy | Rarely felt good about being a boy | Never felt good about being a boy |
| --- | --- | --- | --- | --- |

1. As a child did you ever enjoy wearing dresses and other clothes that girls wear? If so, how often did you enjoy that?

| Always enjoyed wearing dresses and other clothes that girls wear | Usually enjoyed wearing dresses and other clothes that girls wear | Sometimes enjoyed wearing dresses and other clothes that girls wear | Rarely enjoyed wearing dresses and other clothes that girls wear | Never enjoyed wearing dresses and other clothes that girls wear |
| --- | --- | --- | --- | --- |

**Femininity/masculinity (present)**

How do you see yourself in terms of masculinity and femininity? Masculine refers to men or women who feel, look and act like “real” men or in a manner which most people think that men should be like. Feminine is the opposite of masculine and refers to what usually is expected from women. People, men or women, who look and behave like “real” women are called feminine. Indicate on a scale from 1 (not at all) to 5 (extremely) how masculine and feminine you think you are.

1. In general, how masculine do you think you are?

| Not at all | Very little | Fairly | Very much | Extremely |
| --- | --- | --- | --- | --- |

1. In general, how masculine do you act and behave?

| Not at all | Very little | Fairly | Very much | Extremely |
| --- | --- | --- | --- | --- |

1. How masculine do you think you appear and come across to others?

| Not at all | Very little | Fairly | Very much | Extremely |
| --- | --- | --- | --- | --- |

1. In general, how feminine do you think you are?

| Not at all | Very little | Fairly | Very much | Extremely |
| --- | --- | --- | --- | --- |

1. In general, how feminine do you act and behave?

| Not at all | Very little | Fairly | Very much | Extremely |
| --- | --- | --- | --- | --- |

1. How feminine do you think you appear and come across to others?

| Not at all | Very little | Fairly | Very much | Extremely |
| --- | --- | --- | --- | --- |

**Sexual identity confusion**

For each of the following statements, mark the response that best indicates your experience as a gay, or bisexual person. Please be as honest as possible in your responses.

1. I am not totally sure what my sexual orientation is.

| *Disagree strongly* | Disagree | Agree | Agree strongly |
| --- | --- | --- | --- |

1. I keep changing my mind about my sexual orientation.

| *Disagree strongly* | Disagree | Agree | Agree strongly |
| --- | --- | --- | --- |

1. I can't decide whether I am bisexual, homosexual, or heterosexual.

| *Disagree strongly* | Disagree | Agree | Agree strongly |
| --- | --- | --- | --- |

1. I get very confused when I try to figure out my sexual orientation.

| *Disagree strongly* | Disagree | Agree | Agree strongly |
| --- | --- | --- | --- |

**Internalized homophobia**

Do you agree or disagree with the following statements:

1. Sometimes I dislike myself for being a man who has sex with other men.

| *Disagree strongly* | Disagree | Agree | Agree strongly |
| --- | --- | --- | --- |

1. I wish I were only sexually attracted to women.

| *Disagree strongly* | Disagree | Agree | Agree strongly |
| --- | --- | --- | --- |

1. I have tried to become more sexually attracted to women.

| *Disagree strongly* | Disagree | Agree | Agree strongly |
| --- | --- | --- | --- |

1. I am ashamed of myself for being sexually attracted to other men.

| *Disagree strongly* | Disagree | Agree | Agree strongly |
| --- | --- | --- | --- |

1. I am comfortable with my sexual attraction to other men.

| *Disagree strongly* | Disagree | Agree | Agree strongly |
| --- | --- | --- | --- |

1. I feel that being attracted to men is a personal weakness for me.

| *Disagree strongly* | Disagree | Agree | Agree strongly |
| --- | --- | --- | --- |

1. If someone offered me the chance to be completely heterosexual, I would accept the chance.

| *Disagree strongly* | Disagree | Agree | Agree strongly |
| --- | --- | --- | --- |

**Secretiveness about sexuality**

1. How many people in your family know that you are sexually attracted to persons of the same sex?

| *None of them* | Some of them | Most of them | All of them |
| --- | --- | --- | --- |

1. How many of your friends know that you are sexually attracted to men?

| *None of them* | Some of them | Most of them | All of them |
| --- | --- | --- | --- |

1. How many people in your township/community know that I am sexually attracted to persons of the same sex?

| *None of them* | Some of them | Most of them | All of them |
| --- | --- | --- | --- |

1. In general, how hard do you try to keep your sexual orientation hidden from your family?

| Try very hard | Try somewhat hard | Don’t try, but don’t talk about it | I openly talk about it with my family | Not applicable |
| --- | --- | --- | --- | --- |

1. In general, how hard do you try to keep your sexual orientation hidden from your friends?

| Try very hard | Try somewhat hard | Don’t try, but don’t talk about it | I openly talk about it with my family | Not applicable |
| --- | --- | --- | --- | --- |

1. In general, how hard do you try to keep your sexual orientation hidden at school, college or university?

| Try very hard | Try somewhat hard | Don’t try, but don’t talk about it | I openly talk about it with my family | Not applicable |
| --- | --- | --- | --- | --- |

1. In general, how hard do you try to keep your sexual orientation hidden at work?

| Try very hard | Try somewhat hard | Don’t try, but don’t talk about it | I openly talk about it with my family | Not applicable |
| --- | --- | --- | --- | --- |

1. In general, how hard do you try to keep your sexual orientation hidden when you are in public spaces?

| Try very hard | Try somewhat hard | Don’t try, but don’t talk about it | I openly talk about it with my family | Not applicable |
| --- | --- | --- | --- | --- |

**Gender-based discrimination**

In the past year how often have the following things happened to you:

1. …had verbal insults and curses directed at you?

| *Never* | Rarely | Sometimes | Often |
| --- | --- | --- | --- |

1. …been threatened with physical violence?

| *Never* | Rarely | Sometimes | Often |
| --- | --- | --- | --- |

1. …had your personal possessions damaged or destroyed?

| *Never* | Rarely | Sometimes | Often |
| --- | --- | --- | --- |

1. …had objects thrown at you?

| *Never* | Rarely | Sometimes | Often |
| --- | --- | --- | --- |

1. …been chased or followed?

| *Never* | Rarely | Sometimes | Often |
| --- | --- | --- | --- |

1. …been spat on?

| *Never* | Rarely | Sometimes | Often |
| --- | --- | --- | --- |

1. …been punched, hit, kicked, or beaten?

| *Never* | Rarely | Sometimes | Often |
| --- | --- | --- | --- |

1. … been assaulted or wounded with a weapon?

| *Never* | Rarely | Sometimes | Often |
| --- | --- | --- | --- |

1. … been sexually harassed or abused (not raped)?

| *Never* | Rarely | Sometimes | Often |
| --- | --- | --- | --- |

1. … been raped?

| *Never* | Rarely | Sometimes | Often |
| --- | --- | --- | --- |

**AUDIT-C**

1. How often do you have a drink containing alcohol?

| Never | Monthly or less | 2-4 times per month | 2-3 times per week | 4+ times per week |
| --- | --- | --- | --- | --- |

1. How many drinks containing alcohol do you have on a typical day when you are drinking?

| 1 or 2 | 3 or 4 | 5 or 6 | 7-9 | 10+ |
| --- | --- | --- | --- | --- |

1. How often do you have six or more drinks on one occasion?

| Never | Less than monthly | Monthly | Weekly | Daily or almost daily |
| --- | --- | --- | --- | --- |

**Boredom**

How much do you agree or disagree with the following statements:

1. I feel bored most of the time.

| *Disagree strongly* | Disagree | Agree | Agree strongly |
| --- | --- | --- | --- |

1. What I do in my free time makes me happy.

| *Disagree strongly* | Disagree | Agree | Agree strongly |
| --- | --- | --- | --- |

1. It's difficult to find interesting things to do in my free time

| *Disagree strongly* | Disagree | Agree | Agree strongly |
| --- | --- | --- | --- |

1. I enjoy the free time that I have.

| *Disagree strongly* | Disagree | Agree | Agree strongly |
| --- | --- | --- | --- |

1. I don’t know how to spend my free time.

| *Disagree strongly* | Disagree | Agree | Agree strongly |
| --- | --- | --- | --- |

**Hopelessness**

1. In the future, I expect to succeed in what concerns me most.

| *Disagree strongly* | Disagree | Agree | Agree strongly |
| --- | --- | --- | --- |

1. My future seems bleak to me.

| *Disagree strongly* | Disagree | Agree | Agree strongly |
| --- | --- | --- | --- |

1. I just cannot get the breaks, and there’s no reason I will in the future.

| *Disagree strongly* | Disagree | Agree | Agree strongly |
| --- | --- | --- | --- |

1. I have great faith in the future.

| *Disagree strongly* | Disagree | Agree | Agree strongly |
| --- | --- | --- | --- |

**Self-esteem and Self-confidence**

1. I have high self-esteem.

| *Disagree strongly* | Disagree | Agree | Agree strongly |
| --- | --- | --- | --- |

1. I am a very confident person.

| *Disagree strongly* | Disagree | Agree | Agree strongly |
| --- | --- | --- | --- |

1. My life is going into the right direction.

| *Disagree strongly* | Disagree | Agree | Agree strongly |
| --- | --- | --- | --- |

**Happiness**

1. Do you in general consider yourself as a happy or unhappy person?

| *Very unhappy* | Unhappy | Happy | Very happy |
| --- | --- | --- | --- |

1. Compared to most of your peers, do your consider yourself as more or less happy?

| *Much less happy* | Less happy | More happy | Much more happy |
| --- | --- | --- | --- |

**Depression and Anxiety**

1. Over the last 2 weeks, how often have you had little interest or pleasure in doing things.

| Not at all | Several days | More than half the days | Nearly everyday |
| --- | --- | --- | --- |

1. Over the last 2 weeks, how often have you felt down, depressed, or hopeless.

| Not at all | Several days | More than half the days | Nearly everyday |
| --- | --- | --- | --- |

1. In the last 4 weeks, have you had an anxiety attack ― suddenly feeling fear or panic?

| Yes | No |
| --- | --- |

1. If yes, has this ever happened before?

| Yes | No |
| --- | --- |
